# Aleukemic Leukemia Cutis: An Unusual Rash in a Child

**DOI:** 10.4274/Tjh.2013.0019

**Published:** 2014-06-10

**Authors:** Didem Atay, Emine Türkkan, Kübra Bölük

**Affiliations:** 1 Okmeydani Education and Research Hospital, Department of Pediatric Hematology and Oncology, İstanbul, Turkey

**Keywords:** Leukemia cutis, Aleukemic leukemia, Persistent

## 3. CLINICAL PICTURE IN HEMATOLOGY

**Figure 1:**
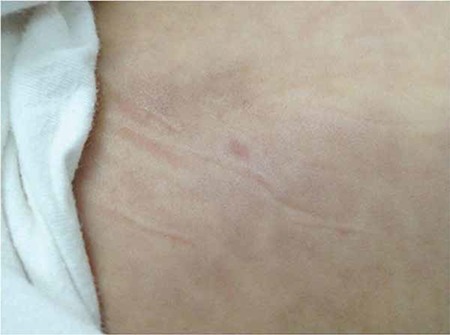
Erythematous macules on the abdomen.

**Figure 2:**
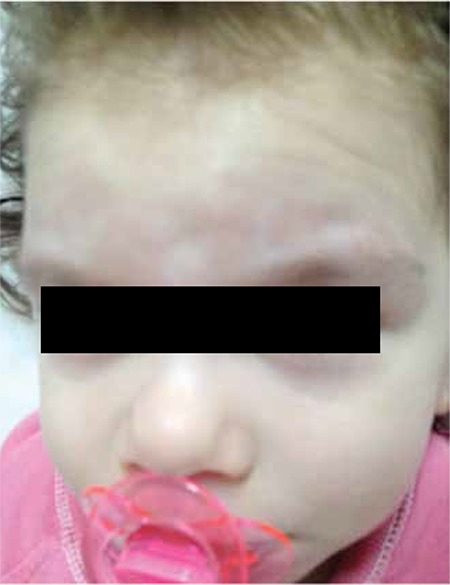
Tumor with no signs of inflammation on brow area.

**Figure 3:**
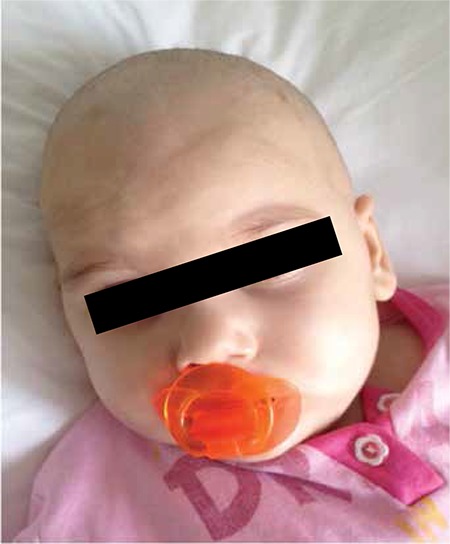
Regression of the soft tissue tumor.

A previously healthy 2-year-old girl presented with a rash of erythematous macules on the abdomen (Figure 1). In 2 weeks the rash spread to her back and brows and became violaceous. She had a firm, non-fluctuant, non-tender, and smooth-surfaced tumor with no signs of inflammation on her brow area (Figure 2). She had no signs or symptoms except for a mild fever for 10 days. A complete blood count and peripheral blood smear did not show significant findings. Lactate dehydrogenase was elevated to 873 U/L. The skin lesions were treated with a combination of antihistamines and topical steroids without response. A skin biopsy performed 4 weeks after the onset of rash showed leukemic blast cells (B-cell lymphoblastic lymphoma/leukemia). A bone marrow aspirate confirmed precursor B-cell acute lymphoblastic leukemia with 30% blast cells. Upon immunohistochemical analysis, the neoplastic cells were positive for CD10, CD19, CD22, HLA-DR, and terminal deoxynucleotidyl transferase (TdT). Cytogenetic studies showed normal 46XX karyotype and ALL chromosomal translocations were negative. Magnetic resonance examination of the cranium revealed enlargement of the frontal soft tissue in the brow area, without evidence of bone involvement. Induction therapy was started immediately using protocol ALL IC BFM 2009. On the 10th day of induction chemotherapy, the soft tissue and skin lesions had almost completely regressed (Figure 3). 

Leukemia cutis is an infiltration of the skin by neoplastic leukocytes. Although leukemia cutis tends to present with other features of leukemia, it can occasionally precede the development of blast cells in the marrow and blood (aleukemic leukemia cutis) [1]. In childhood, it is seen more commonly in congenital leukemia and acute myelogenous leukemia (10%) than in pediatric acute lymphoblastic leukemia (1%). It is generally treated with ALL-type regimens, which are associated with a favorable outcome in more than 70% of cases when started early [2,3,4]. In conclusion, it is important to know that the disease must be treated as a diagnostic and therapeutic emergency.
